# Activation of the pre-supplementary motor area but not inferior prefrontal cortex in association with short stop signal reaction time – an intra-subject analysis

**DOI:** 10.1186/1471-2202-10-75

**Published:** 2009-07-14

**Authors:** Herta HA Chao, Xi Luo, Jeremy LK Chang, Chiang-shan R Li

**Affiliations:** 1 Department of Internal Medicine, VA Connecticut Health Care System, West Haven, CT 06516, USA; 2 Department of Internal Medicine, Yale University School of Medicine, New Haven, CT 06519, USA; 3 Department of Psychiatry, Yale University School of Medicine, New Haven, CT 06519, USA; 4 Department of Statistics, Yale University, New Haven, CT 06519, USA; 5 Department of Neurobiology, Yale University School of Medicine, New Haven, CT 06520, USA

## Abstract

**Background:**

Our previous work described the neural processes of motor response inhibition during a stop signal task (SST). Employing the race model, we computed the stop signal reaction time (SSRT) to index individuals' ability in inhibitory control. The pre-supplementary motor area (preSMA), which shows greater activity in individuals with short as compared to those with long SSRT, plays a role in mediating response inhibition. In contrast, the right inferior prefrontal cortex (rIFC) showed greater activity during stop success as compared to stop error. Here we further pursued this functional differentiation of preSMA and rIFC on the basis of an intra-subject approach.

**Results:**

Of 65 subjects who participated in four sessions of the SST, we identified 30 individuals who showed a difference in SSRT but were identical in other aspects of stop signal performance between the first ("early") and last two ("late") sessions. By comparing regional brain activation between the two sessions, we confirmed greater preSMA but not rIFC activity during short as compared to long SSRT session within individuals. Furthermore, putamen, anterior cerebellum and middle/posterior cingulate cortex also showed greater activity in association with short SSRT.

**Conclusion:**

These results are consistent with a role of medial prefrontal cortex in controlled action and inferior frontal cortex in orienting attention. We discussed these findings with respect to the process of attentional monitoring and inhibitory motor control during stop signal inhibition.

## Background

Response inhibition allows flexible motor acts in changing environment. The stop signal task (SST) has been widely used to investigate the behavioral and neural processes of motor response inhibition [1,2]. In the SST, there are "go" and "stop" trials. In the go trials, participants are required to respond to an imperative stimulus within a time window. Because go trials occur most of the time, they set up a prepotent response tendency. In the stop trials, an additional stop signal instructs participants to withhold their response. The rationale is that, when response inhibition is in place, participants are able to stop upon seeing the stop signal. Thus, many previous studies have compared stop success with stop error trials and identified bilateral or right inferior prefrontal cortex (IFC) as a cortical site of inhibitory motor control [3-5] (see also [6] for a review). However, an extensive literature has suggested that the IFC is part of the ventral attention system [7,8]. Thus, by increasing activity in response to the stop signal (a behaviorally relevant external stimulus), the IFC may serve to orient attention and processing resources including those related to inhibitory control to the stop process and, as a result, facilitate stop signal inhibition.

In order to isolate the neural correlates independent of such attention-related activity, we proposed to follow the race model and computed the stop signal reaction time (SSRT) for individual subjects [1,9]. By comparing participants with short and long SSRT who otherwise were indistinguishable in stop signal performance, we isolated the anterior pre-supplementary motor area (preSMA) as a potential cortical locus of response inhibition [9]. However, this between-subject approach is amenable to demographic and behavioral confounds that go beyond the SST. For instance, impulsivity as a personality trait is known to influence cerebral activity during cognitive and affective processing [10,11]. Furthermore, in a different cognitive control task, Forstmann and colleagues demonstrated that inter-subject variation in reaction time could provide useful information in model-based analyses of fMRI data, suggesting that an important dimension of inter-subject variability may elude "traditional" general linear modeling [12]. Therefore, in order to avert potential confounds associated with such and other inter-subject factors, we attempted to confirm the role of pre-SMA in motor response inhibition on the basis of a within-subject approach. We took advantage of the multiple-session data that we have collected in healthy volunteers and compared regional brain activation between sessions in which participants showed a difference in SSRT. Importantly, participants did not differ in other performance measures between these short and long SSRT sessions. To anticipate, we replicated the finding of greater preSMA activity in association with short as compared to long SSRT.

## Methods

### Subjects and behavioral task

Sixty-five subjects (22 to 48 years of age, all right-handed, 32 men) were paid to participate in the study. All subjects signed a written consent after details of the study were explained, in accordance to institute guidelines and procedures approved by the Yale Human Investigation Committee. The study was carried out in compliance with the Helsinki Declaration.

**Figure 1:**
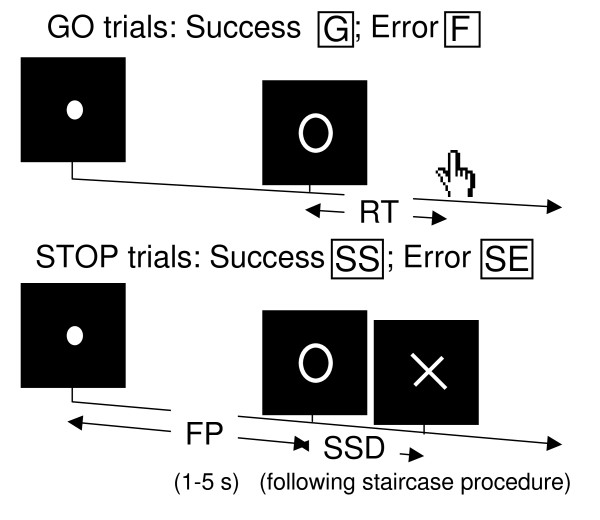
**Stop signal paradigm: In "go" trials (75%) observers responded to the go signal (a circle) and in "stop" trials (25%) they had to withhold the response when they saw the stop signal (an X)**. In both trials the go signal appeared after a randomized time interval between 1 to 5 s (the fore-period or FP, uniform distribution) following the appearance of the fixation point. The go signal disappeared at the time of button press or when 1 s had elapsed, whichever came first, ending the trial. In a stop trial, the stop signal replaced the go signal by a time delay – the stop signal delay (SSD). The SSD was updated according to a staircase procedure, whereby it increased and decreased by 64 ms following a stop success and stop error trial, respectively.

We employed a simple reaction time task in this stop-signal paradigm [9,13] (Figure 1). There were two trial types: "go" and "stop," randomly intermixed. A small dot appeared on the screen to engage attention and eye fixation at the beginning of a go trial. After a randomized time interval (fore-period) anywhere between 1 and 5 s, the dot turned into a circle (the "go" signal), prompting subjects to quickly press a button. The circle vanished at button press or after 1 s had elapsed, whichever came first, and the trial terminated. A premature button press prior to the appearance of the circle also terminated the trial. Three quarters of all trials were go trials. The remaining one quarter were stop trials, in which an additional "X," the "stop" signal, appeared after and replaced the go signal. Subjects were told to withhold button press upon seeing the stop signal. Likewise, a trial terminated at button press or when 1 s had elapsed since the appearance of the stop signal. The SSD (stop signal delay) – the time interval between go and stop signal onsets – started at 200 ms and varied from one stop trial to the next according to a staircase procedure: if the subject succeeded in withholding the response, the SSD increased by 64 ms; conversely, if they failed, SSD decreased by 64 ms [14,15]. There was an inter-trial-interval of 2 s. Subjects were instructed to respond to the go signal quickly while keeping in mind that a stop signal could come up in a small number of trials. Prior to the fMRI study each subject had a practice session outside the scanner. In the scanner each subject completed four 10-min sessions of the task with the SSD updated manually across sessions. Depending on the actual stimulus timing (trial varied in fore-period duration) and speed of response, the total number of trials varied slightly across subjects in an experiment. With the staircase procedure we anticipated that the subjects succeeded in withholding their response in approximately half of the stop trials.

The staircase procedure was also important for us to apply the race model in the computation of stop signal reaction time (SSRT) as an index of motor response inhibition [1,2]. One way to understand the stop signal task is in terms of a race model with the go and stop processes racing toward a finishing line (Logan, 1994). The go process prepares and generates the movement while the stop process inhibits movement initiation: whichever process finishes first determines whether a response is initiated or not. Importantly, the go and stop processes race toward the activation threshold independently. Thus, the time required for the stop signal to be processed so a response is withheld (SSRT) can be computed on the basis of the go trial RT distribution and the odds of successful inhibits for different time delays between go and stop signals. This is done by estimating the critical SSD at which a response can be correctly stopped in approximately 50% of the stop trials. With the assumptions of this "horse-race" model, the SSRT could then be computed for each individual subject by subtracting the critical SSD from the median go trial RT. Generally speaking, the SSRT is the time required for a subject to cancel the movement after seeing the stop signal. A long SSRT indicates poor response inhibition.

In a within-subject analysis, we combined data of session 1 and 2 into an "early" session and data of session 3 and 4 into a "late" session. Because of the temporal contiguity of session 1 and 2, and session 3 and 4, we were able to examine whether participants' performance was adequate so we could compute the SSRT each for the early and late session. Thirty subjects showed a go trial success rate greater than 95% and a stop trial success rate within 50 ± 5%, suggesting that their performance is adequately tracked by the staircase procedure, during *both *early and late sessions. We estimated separately for the early and late session a critical SSD that represents the time delay between go and stop signals that a subject would require in order to succeed in 50% of the stop trials [15]. Specifically, SSDs across trials were grouped into runs, with each run being defined as a monotonically increasing or decreasing series. We derived a mid-run estimate by taking the middle SSD (or average of two middle SSDs if there was an even number of SSDs) of every second run. The critical SSD was computed by taking the mean of all mid-run SSDs. It was reported that, except for experiments with a small number of trials (less than 30), the mid-run estimate was close to the maximum likelihood estimate of X_50 _(50% positive response; i.e., 50% SS in the SST, [16]). The SSRT was computed for individual subjects by subtracting the critical SSD from the median go trial RT each for the early and late session [1,15].

### Imaging protocol

Conventional T_1_-weighted spin echo sagittal anatomical images were acquired for slice localization using a 3T scanner (Siemens Trio). Anatomical images of the functional slice locations were next obtained with spin echo imaging in the axial plane parallel to the Anterior commissure – posterior commissure line with repetition time = 300 ms, echo time = 2.5 ms, bandwidth = 300 Hz/pixel, flip angle = 60°, field of view = 220 × 220 mm, matrix = 256 × 256, 32 slices with slice thickness = 4 mm and no gap. Functional, blood oxygenation level dependent (BOLD) signals were then acquired with a single-shot gradient echoplanar imaging (EPI) sequence. Thirty-two axial slices parallel to the AC-PC line covering the whole brain were acquired with TR = 2,000 ms, TE = 25 ms, bandwidth = 2004 Hz/pixel, flip angle = 85°, field of view = 220 × 220 mm, matrix = 64 × 64, 32 slices with slice thickness = 4 mm and no gap. Three hundred images were acquired in each session for a total of 4 sessions.

### Data analysis and statistics

Data were analyzed with Statistical Parametric Mapping version 5 (Wellcome Department of Imaging Neuroscience, University College London, U.K.). Images from the first five TRs at the beginning of each trial were discarded to enable the signal to achieve steady-state equilibrium between RF pulsing and relaxation. Images of each individual subject were first corrected for slice timing, realigned (motion-corrected) and unwarped [17,18]. A mean functional image volume was constructed for each subject for each session from the realigned image volumes. These mean images were normalized to an MNI (Montreal Neurological Institute) EPI template with affine registration followed by nonlinear transformation [19,20]. The normalization parameters determined for the mean functional volume were then applied to the corresponding functional image volumes for each subject. Finally, images were smoothed with a Gaussian kernel of 10 mm at Full Width at Half Maximum. The data were high-pass filtered (128 s cutoff) to remove low-frequency signal drifts.

Four main types of trial outcome were first distinguished: go success (G), go error (F), stop success (SS), and stop error (SE) trial. A statistical analytical design was constructed each of the early and late session for individual subjects, using the general linear model (GLM) with the onsets of go signal in each of these trial types convolved with a canonical hemodynamic response function (HRF) and with the temporal derivative of the canonical HRF and entered as regressors in the model [21]. Realignment parameters in all 6 dimensions were also entered in the model. Serial autocorrelation was corrected by a first-degree autoregressive or AR(1) model. The GLM estimated the component of variance that could be explained by each of the regressors.

In the first-level analysis, we constructed for individual subjects a contrast between SS and SE each for the early and late session. The *con *or contrast (difference in β) images of the first-level analysis were then used for the second-level group statistics (random effect analysis; [22]). Brain regions were identified using an atlas [23]. Because of our hypothesis targeting the pre-supplementary motor area (preSMA) and right inferior prefrontal cortex (rIFC), we employed small volume correction to examine results specifically for these structures. The preSMA (MNI coordinate: x = -4, y = 36, z = 56) and rIFC (x = 44, y = 48, z = -12) masks were derived from our published work [9]. In addition, we derived a second rIFC mask (a sphere of 10 mm in radius centered at x = 44, y = 12, z = 8) based on Aron and Poldrack, which reported a slightly different location of activation. All templates were in MNI space and voxel activations were presented in MNI coordinates. We used MarsBaR to derive for each individual subject the effect size of SS>SE for the ROIs [24].

## Results

### Stop signal performance

Table 1 shows the results of behavioral performance, separately for sessions with short and long SSRT. Note that our subjects succeeded in approximately 50% of the stop trials, suggesting the success of the tracking procedure in each of the two sessions. Subjects were also indistinguishable in go success rate and median go trial RT between the two sessions. We also computed a fore-period (FP) effect to index the extent of motor preparation. With the FP randomly varying between 1 and 5 s, we compared the RT of go trials with a FP less than 3 s and those with a FP equal to or greater than 3 s [25,26]. The effect size of the FP effect did not differ between the short and long SSRT sessions. Furthermore, we computed RT difference between go trials following a stop error and those following a go success, to index performance monitoring during the task [9,13,27,28]. This post-error slowing (PES) effect also did not differ between the two sessions. Thus, the two sessions differed in SSRT but not any other aspects of stop signal performance.

**Table 1:** Behavioral performance in the stop signal task, grouped by SSRT and "early" versus "late" session

**SSRT, session**	**SSRT** **(ms)**	**Median go** **RT (ms)**	**%go**	**%stop**	**PES effect** **(effect size)**	**FP** **(effect size)**
Short	180 ± 29	522 ± 122	98.3 ± 1.7	50.0 ± 3.2	1.18 ± 1.68	2.31± 1.34
Short, early	178 ± 27	504 ± 101	98.6 ± 1.6	51.5 ± 2.1	1.34 ± 1.20	1.81 ± 1.29
Short, late	181 ± 31	539 ± 139	98.0 ± 1.7	48.7 ± 3.4	1.04 ± 1.79	2.75 ± 1.25
Long	226 ± 29	529 ± 120	98.4 ± 1.3	50.3 ± 3.3	1.23 ± 1.62	2.32 ± 1.20
Long, early	235 ± 26	514 ± 130	98.5 ± 1.3	51.4 ± 3.4	1.14 ± 1.52	2.51 ± 1.25
Long late	216 ± 30	546 ± 110	98.2 ± 1.4	49.1 ± 2.8	1.34 ± 1.71	2.11 ± 1.14
P value^a^	<0.0001	0.637	0.713	0.731	0.874	0.976
P value^b^	<0.0001	0.484	0.793	0.870	0.884	0.927

Note: SSRT: stop-signal reaction time; %go and %stop: percentage of successful go and stop trials; FP: fore-period; PES: post-error slowing; *effect size; all values are mean ± standard deviation; ^a^P value based on paired sample t test (short vs. long SSRT); ^b^P value for the within-subject contrast based on a repeated measure ANVOA with SSRT as a within-subject variable and group as a between-subject variable. See text for further explanation.

Because 16 subjects showed long SSRT during the early session (as compared to the late session) and 14 subjects showed the reverse, we needed to account for a potential "order" effect in the behavioral data. To this end, we performed a repeated measure analysis of variance (ANOVA) with SSRT (short vs. long) session as a within-subject variable and group (early vs. late session with short SSRT) as a between-subject variable. The results again showed no differences other than in SSRT between the short and long SSRT sessions (Table 1).

### Region of interest analyses

**Figure 2:**
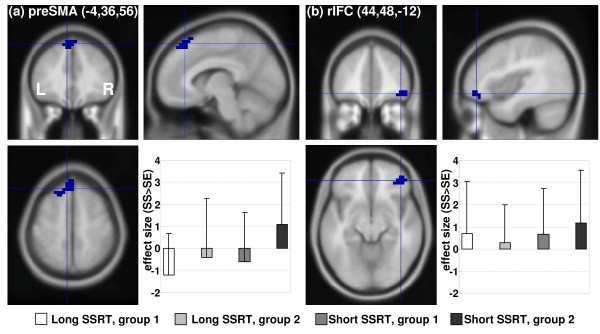
**Effect sizes of stop signal inhibition in association with stop signal reaction time (SSRT)**: (a) Pre-supplementary motor area (preSMA) and effect size (mean ± standard deviation) of stop success (SS) > stop error (SE) for short and long SSRT sessions and subject groups; group 1 = long SSRT during "early" sessions; group 2 = short SSRT during "early" sessions; (b) Right inferior prefrontal cortex (rIFC) and effect size (mean ± standard deviation) of SS > stop error SE for short and long SSRT sessions and subject groups.

To test our hypothesis, we compared short and long SSRT sessions with a paired sample t test with small volume correction for the preSMA and rIFC masks. The results showed greater activity in the preSMA in the short as compared to long SSRT sessions (p < 0.020, corrected for family-wise error of multiple comparison, x = 0, y = 40, z = 56; voxel Z = 2.96). In contrast, neither rIFC mask differentiated between short and long SSRT sessions at a threshold of p = 0.05, uncorrected. Likewise, because of potential "order" effect, we compared the effect size of stop success > stop error each for preSMA and rIFC masks on the basis of a 2-factor repeated measure ANOVA with SSRT (short vs. long) session as a within-subject variable and group (early vs. late session with short SSRT) as a between-subject variable. The results showed greater activity in the preSMA (F_1,28 _= 4.843, p = 0.030) but not in the rIFC mask (F_1,28 _= 0.808 and 0.916, p = 0.376 and 0.371) in the short as compared to long SSRT session (within-subject factor). Figure 2 illustrated these results for preSMA and one rIFC mask.

### Whole brain analyses

**Figure 3:**
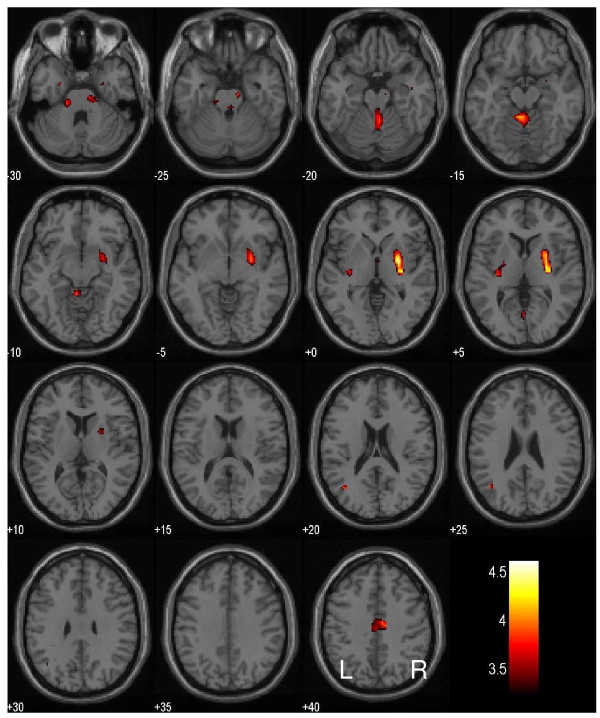
**Regional brain activation in short as compared to long stop signal reaction time (SSRT)**: BOLD activations in the putamen, middle/posterior cingulate cortex and cerebellum in association with short as compared to long SSRT. Color bar indicates voxel T value.

To identify potential new brain regions with this intra-subject analysis, we compared short and long SSRT sessions for the whole brain using a paired sample t test. The results showed that, compared to long SSRT, short SSRT session was associated with greater activation in the right putamen (x = 28, y = -4, z = 0, voxel Z = 4.24, 5,184 mm^3^), the medial aspect of the central lobule in the anterior cerebellum, part of the cerebellar vermis (x = -4, y = -44, z = -12, voxel Z = 4.09, 3,008 mm^3^) and the middle/posterior cingulate cortex (x = 8, y = -12, z = 40, voxel Z = 3.74, 1,792 mm^3^), at a threshold of p < 0.001, uncorrected, and 20 voxels in the extent of activation (Figure 3). In contrast, no brain regions showed greater activation during the long as compared to the short SSRT session.

We conducted a SSRT (short vs. long) session by group (early vs. late session with short SSRT) mixed effect ANOVA, in order to account for the order effect. The results showed activity in the same brain regions but with diminished significance (p < 0.01, uncorrected, and 10 voxels in extent of activation), including right putamen: x = 28, y = -4, z = 0, voxel Z = 3.09; 3,648 mm^3^; anterior cerebellum: x = -4, y = -44, z = -12, voxel Z = 2.73, 1,472 mm^3^; and middle/posterior cingulate cortex: x = 8, y = -16, z = 40, voxel Z = 2.56, 704 mm^3^.

## Discussion

The stop signal task (SST) is widely used to investigate the behavioral and neural processes of motor inhibitory control [1,2]. In studies employing fixed stop signal delays (SSD), an inhibitory function could be computed from stop success rate at these SSDs to index individuals' ability of inhibitory control. The inhibitory function or the stop signal reaction time (SSRT) computed from the inhibitory function can then be used in, for example, comparing patients and healthy controls (see [29] for a review) or tracking the development of inhibitory control through adolescence [30,31]. In studies employing a staircase procedure (as in the current study), the SSRT can be computed directly for this purpose. Thus, in behavioral SST studies, there is much consistency as to what represents the outcome measure of inhibitory control.

The imaging literature presents a slightly complicated picture [6]. For instance, many studies have compared stop and go trials directly in order to identify the neural processes of response inhibition [3,32-34]. The rationale for this contrast perhaps is that the stop but not go process involves response inhibition. Compared to go trials, however, stop trials evoked more perceptual processing. Furthermore, response inhibition is not invariably evoked during stop trials, and subjects succeed or fail in inhibitions depending on whether this capacity is in place. Comparing stop and go trials without distinguishing stop success and error seems to be inconsistent with the underlying rationale of the SST. Other studies contrasted stop success and errors to isolate inhibitory control [4,5]. However, successful performance in the SST depends on a number of other cognitive processes in addition to response inhibition. For instance, if participants watch for the stop signal, it is likely that this attention would expedite stop signal processing and facilitate the initiation of motor response inhibition.

We followed the race model by using the SSRT as an index of inhibitory control and identified the anterior pre-supplementary motor area (preSMA) as a brain region mediating response inhibition by comparing individuals with short and long SSRT [9]. Thus, we replicated here greater activity in the preSMA in association with short SSRT on the basis of a within-subject analysis. In contrast, the inferior frontal cortices did not differentiate between short and long SSRT. These results stood when the order effect of the SSRT sessions was accounted for. We thus confirmed our hypothesis that this medial prefrontal area supports a role of inhibitory control, in keeping with its function in action selection and cognitive control shown in the literature [3,35-49]. In contrast, the IFC is likely to be involved in attentional monitoring and allocation of processing resources, "kicking start" the stop process. Interestingly, consistent with bilateral IFC activation during stop success as compared to stop error [4,5,9], a recent work showed increased no-go errors in patients with left IFC lesions [50].

In whole brain analyses, we identified at a moderate threshold three additional structures related to stop signal inhibition, which were not observed in between-subject analyses: right putamen, middle/posterior cingulate cortex (PCC) and part of the vermis in the anterior cerebellum. The finding of right putamen activity in association with short SSRT directly contradicted our previous report which showed greater putamen activity in subjects with long compared to short SSRT [51]. In fact, a recent study suggested that putamen is a target of prefrontal cortical action of motor inhibition [52]. On the other hand, putamen lesion is known to cause apraxia, a loss of ability in goal-directed movement [53]. Putamen is involved in the timing of sequential movements [54]. In a unilateral motor task, the putamen in the ipsilateral hemisphere coactivated more strongly with the controlling motor cortex (contralateral to movement) than with the noncontrolling cortex, suggesting a complex role of putamen in motor control and its dependence on hemispherity [55]. Studies also documented activity in the putamen and the cerebellar vermis in movement that requires bimanual coordination [56]. Both putamen and cerebellum showed greater activation when participants were engaged in spatially incompatible (between hands) drawing than in spatially compatible drawing while the primary motor cortex showed the opposite pattern of response [57]. Taken together, these studies suggested a role of the putamen in the control rather than simple execution of movement.

The PCC has been implicated in functional neuroimaging in a wide variety of cognitive and affective processes and in the pathogenesis of many neurological conditions [58]. Although no studies have to our knowledge suggested a specific role of PCC in inhibitory control, a few earlier findings could be discussed with the current result. For instance, a recent magnetoencephalographic study illustrated the importance of enhanced perceptual processing of the stop stimulus in stop signal inhibition [59]. In particular, the PCC appeared to be a critical site where this enhancement occurs and, via its interconnections with prefrontal structures, greater PCC activity facilitates stop signal inhibition [59]. Other studies implicated a role of the PCC in motor response inhibition in different behavioral paradigms [60,61]. For instance, in an attention cueing task, a spatial cue evoked greater activity in cortical structures including the retrosplenial PCC at a time when premature movement had to be withheld before the target appeared [60]. Also of note is that the cingulate brain region we identified here did not appear to extend posteriorly to involve the retrosplenial cortex. Further studies were warranted to examine the specific roles of this cingulate region in motor inhibitory control.

We identified in association with short as compared to long SSRT the central lobule of the anterior cerebellum, part of the cerebellar vermis, a structure known to be important for motor control [62]. In particular, cerebellum activates in response to timed movement which presumably involved a greater extent of inhibitory control, as compared to response to movement guided by external stimulus [63-66]. Similarly, non-predictive but not learned, predictable sequence of movement appeared to activate the vermis [67], potentially because the former required more moment-to-moment control. It has been suggested that cerebellar vermis is important for us to understand the neural processes underlying a number of psychiatric conditions, including attention deficit hyperactivity disorder [68-70], schizophrenia [71-73], bipolar disorder [74,75], and cocaine [76] and alcohol [77-80] use disorders, in which deficits in inhibitory control are implicated. Thus, the finding of greater cerebellar activation in association with response inhibition may also be relevant to studies of these clinical conditions.

## Conclusion

To summarize, we demonstrated greater activity in the pre-supplementary motor area (pre-SMA) but not the inferior frontal cortex (IFC) within individuals when their short SSRT session were compared to long SSRT session. This within-subjective analysis confirmed a distinct role of the preSMA in mediating stop signal inhibition, as described by the race model. The current findings, along with evidence from eletrophysiological studies [59,81-83], suggest the importance of differentiating attentional monitoring and motor response inhibition in evaluating the neural processes underlying stop signal and go/no-go performance.

The current findings have some limitations. First, the findings on the putamen are inconsistent between the current work and Li et al., 2008 [51]. We have reviewed the literature, which suggested a multifaceted and sometimes contradicting role of the putamen in motor control. However, the latter alone does not explain why opposite findings were obtained for the putamen in the current study. We speculated that inter-subject variability in aspects of the stop signal performance that have not yet been captured by our analyses may have accounted for this discrepancy. Second, although SSRT is generally taken to be a measure of motor inhibitory control [1,2,6,84], a contrast between short and long SSRT may involve cognitive processes other than response inhibition. For instance, the preSMA has been implicated in motor awareness [40]. Given that response inhibition during the stop signal task is likely more "controlled" (as opposed to "automatic" [85]), the role of preSMA in action awareness deserves careful consideration. Furthermore, preSMA along with other prefrontal and parietal structures showed greater activation during attentional shift when switching between target dimensions in stimulus categorization task, suggesting that an attentional component of inhibitory control cannot be ruled out in the current findings [86]. Although it is difficult to directly compare fMRI of humans to unit recording studies in monkeys, a recent report also illustrated the complexity of determining a role of motor inhibitory control in the preSMA [87]. Nakajima and colleagues trained macaque monkeys to perform a sequence of two movements and showed preSMA neuronal activity selective to the second-next movement. Such activity peaked before the initiation of the first movement, decayed thereafter, and remained low in magnitude when the animals initiated the second movement. The authors suggested that, while it could be a signal to inhibit a premature exertion of the second movement, such activity may serve to activate another group of neurons required for planning the second movement [87]. Finally, a recent study appeared to suggest an opposite role of preSMA during decision making in a reaction time task [88]. These authors observed that the preSMA showed greater activation when participants were cued for response speed as compared to accuracy. On the other hand, the location of the preSMA (x = 4, y = 5, z = 45) reported in this study did not seem to overlap the locale (x = -4, y = 36, z = 56) investigated in the current work. Taken together, the role of inhibitory motor control in the preSMA needs to be ascertained in further studies, with attention to possible functional subdivisions within the broad SMA/preSMA area.

## Abbreviations

**ANOVA**: analysis of variance; **BOLD**: blood oxygenation level dependent; **EPI**: echoplanar imaging; **F**: go error; **FP**: fore-period; **G**: go success; **GLM**: general linear model; **HRF**: hemodynamic response function; **IFC**: inferior (pre-)frontal cortex; **MNI**: Montreal Neurological Institute: **PCC**: posterior cingulate cortex; **PES**: post-error slowing; **preSMA**: pre-supplementary motor area; **SE**: stop error; **SS**: stop success; **SSD**: stop signal delay; **SSRT**: stop signal reaction time; **SST**: stop signal task.

## Authors' contributions

HC conceptualized the analysis, analyzed the data and wrote the paper; XL analyzed the data; JC conducted the study; and C-SRL conducted the study, analyzed the data, and wrote the paper. All authors read and approved the final manuscript.
